# Effects of a ketogenic diet on hippocampal plasticity in freely moving juvenile rats

**DOI:** 10.14814/phy2.12411

**Published:** 2015-05-25

**Authors:** J Harry Blaise, David N Ruskin, Jessica L Koranda, Susan A Masino

**Affiliations:** 1 Department of Engineering, Trinity CollegeHartford, Connecticut; 2 Neuroscience Program, Trinity CollegeHartford, Connecticut; 3 Department of Psychology, Trinity CollegeHartford, Connecticut

**Keywords:** Dentate gyrus, ketone bodies, long-term potentiation, paired pulse ratio, synaptic plasticity

## Abstract

Ketogenic diets are low-carbohydrate, sufficient protein, high-fat diets with anticonvulsant activity used primarily as a treatment for pediatric epilepsy. The anticonvulsant mechanism is thought to involve elevating inhibition and/or otherwise limiting excitability in the brain. Such a mechanism, however, might also significantly affect normal brain activity and limit synaptic plasticity, effects that would be important to consider in the developing brain. To assess ketogenic diet effects on synaptic transmission and plasticity, electrophysiological recordings were performed at the perforant path/dentate gyrus synapse in awake, freely-behaving juvenile male rats. Electrodes were implanted 1 week prior to recording. Animals were fed regular chow or a ketogenic diet ad libitum for 3 weeks before recording. Although the ketogenic diet did not significantly alter baseline excitability (assessed by input–output curves) or short-term plasticity (using the paired-pulse ratio), it did reduce the magnitude of long-term potentiation at all poststimulation timepoints out to the last time measured (48 h). The results suggest an effect of ketogenic diet-feeding on the induction magnitude but not the maintenance of long-term potentiation. The lack of effect of the diet on baseline transmission and the paired-pulse ratio suggests a mechanism that limits excitation preferentially in conditions of strong stimulation, consonant with clinical reports in which the ketogenic diet alleviates seizures without a major impact on normal brain activity. Limiting plasticity in a seizure-susceptible network may limit seizure-induced epileptogenesis which may subserve the ongoing benefit of the ketogenic diet in epilepsy.

## Introduction

Ketogenic diets (KDs) are low-carbohydrate, sufficient protein, high-fat diets used to mimic the beneficial antiseizure effects of prolonged fasting as observed historically in epileptic patients. Their therapeutic effect is at least as strong as anticonvulsant drugs (Freeman et al. 2007); in addition, there is evidence they are antiepileptogenic (Muller-Schwarze et al. 1999; Su et al. 2000; Todorova et al. 2000; Hu et al. 2011; Jiang et al. 2012) and effective in adults as well as children (Baborka 1930; Sirven et al. 1999; Bodenant et al. 2008; Mosek et al. 2009; Klein et al. 2010). Hallmark effects of KDs include mildly lowered blood glucose and strongly elevated blood ketone bodies. A number of studies have investigated the effects of KDs on excitability and synaptic plasticity of the rodent hippocampus (Stafstrom et al. 1999; Bough et al. 2003, 2006; Thio et al. 2010; Kawamura et al. 2014; Simeone et al. 2014), a seizure-susceptible structure with clearly distinguished lamellar organization, well-understood circuitry, and well-characterized involvement in learning and memory.

In hippocampal in vitro seizure models, KD feeding prior to electrophysiological recording reduces seizure-like activity (Bough et al. 2003; Kawamura et al. 2014). Likewise, in tissue from genetic or pharmacological epilepsy models, KD feeding reduces seizure-like activity and normalizes various aberrant aspects of synaptic transmission (Stafstrom et al. 1999; Nylen et al. 2008; Simeone et al. 2014). Some studies have specifically implicated elevated activity of inhibitory neurotransmitters and neuromodulators (Nylen et al. 2008; Kawamura et al. 2014). In contrast, KD feeding does not typically affect baseline excitability in the normal hippocampus (Stafstrom et al. 1999; Thio et al. 2000; Masino et al. 2011; Kawamura et al. 2014) (though see Bough et al. 2003), raising the possibility that KD effects might be strongest in hyperexcitable (e.g., epileptic) states. Long-term potentiation (LTP) is a type of synaptic plasticity in which a train or pattern of electrical stimulation produces a reliable long-lasting enhancement of synaptic transmission; this phenomenon in the hippocampus and elsewhere is a likely synaptic substrate for long-term learning and memory (Brown et al. 1990). If KD treatment can modulate LTP, then KDs may also affect learning and memory. Previous work showed a decrease in LTP magnitude as assessed in adult awake behaving rats (Koranda et al. 2011). Given a KD's predominant clinical application in pediatric epilepsy, any effects on baseline synaptic transmission and synaptic plasticity in the developing brain are underexplored. Such effects are important to quantify and consider as KDs and analogous metabolic therapies become increasingly sought after for an increasing array of clinical conditions – including pediatric conditions which may or may not have comorbid seizures – such as autism and Alzheimer's disease.

Here, we characterize synaptic transmission and plasticity, including LTP, in the dentate gyrus of freely behaving juvenile rats fed a control diet or a KD. Because the KD has been most commonly used in the treatment of pediatric epilepsy (Nordli et al. 2001; Zupec-Kania and Spellman 2008), and no reports to date have quantified the effects of a KD in the developing brain in vivo, dietary treatments were started after weaning and recordings were performed in juvenile rats; long-term plasticity was induced by a theta-burst stimulation pattern designed originally to mimic hippocampal activity and produce reliable potentiation (Hyman et al. 2003). Because a dietary modification has complex physiological effects, and at least some of the effects of KDs are generated in the periphery, the KD-induced physiological milieu was maintained by continuing the diet throughout the experiments and by performing all recordings in vivo. For similar reasons, and specifically because acute placement of electrodes disturbs the local microenvironment, recordings were made from electrodes chronically implanted 7 days prior to recording. Finally, because most prior studies in vivo had used anesthetized animals, and the effects of anesthesia could impact baseline synaptic transmission, and particularly synaptic plasticity, all recordings were made in awake, freely moving animals to eliminate any possible anesthesia-related confounds.

## Methods

Male Sprague–Dawley rats, group-housed until after surgery, were assigned diets at weaning at 21 days of age. Cages were fed ad libitum either a KD (F3666; Bio-Serv; with a 6.6:1 ratio of fat-to-(carbohydrate + protein) (Ruskin et al. 2013) or a control diet (CD; LabDiet 5001). Animals were maintained on diets for 14 days before surgery with recording 7 days later at 42 days of age. In previous work with male rats of this age and strain, 2, 10, 19, or 28 days of feeding with this KD significantly elevated blood *β*-hydroxybutyrate and reduced blood glucose (Ruskin et al. 2009, 2013). All protocols were approved by the Trinity College IACUC and were in accordance with the NIH Guide for the Care and Use of Laboratory Animals.

Details of this surgery have been described previously (Blaise and Bronzino 2003). Briefly, anesthetized rats were stereotactically implanted with a chronic concentric bipolar stimulating electrode in the angular bundle (medial perforant path; −6.3 mm posterior from Bregma, 4.0 mm lateral, −2.5 mm dorsoventral) and with a chronic monopolar tungsten recording electrode in the dentate gyrus (−3.0 mm posterior from Bregma, 2.0 mm lateral, −3.3 mm dorsoventral) under electrophysiological control to maximize the evoked field potential. Ground and reference screw electrodes were implanted contralaterally. Electrode wires led to a contact pin headstage fixed to the skull with fast-drying dental acrylic. Recording took place after one recovery week during which diet treatment continued.

Details of recording were described previously (Koranda et al. 2011). Briefly, on the day of recording, rats were placed in a sound-attenuating recording chamber and attached to the recording equipment, allowing free movement. Animals were allowed 1 h to habituate to the recording environment. After verifying an appropriate evoked response, an input/output curve was constructed by averaging 10 population spikes induced by single pulse stimuli at varying intensities. From this input/output curve, the intensity required to evoke 50% of the maximum population spike was determined and used subsequently. Paired-pulse ratios were determined for a range of intervals. Then, a stable baseline of evoked responses to single-pulse stimuli was established for at least 15 min.

To induce LTP, 5 Hz “theta-burst” stimulation pattern (10 bursts of 10 pulses; burst frequency 5 Hz; intraburst pulse frequency 400 Hz) was delivered to the perforant path. Signs of seizure activity during or after the theta-burst stimulation were never observed. Five evoked dentate gyrus population spikes were recorded and averaged at varying times after theta-burst stimulation. Data from eight out of 28 animals were removed from analysis due to (1) input/output curves that were nonmonotonic, or did not reach a clear and reliable maximal asymptote; (2) values more than two standard deviations from the mean during baseline, or (3) signal loss during recording. Significance was determined by ANOVA with Newman-Keuls comparisons. Data are shown as means ± standard error.

## Results

**Figure 1:**
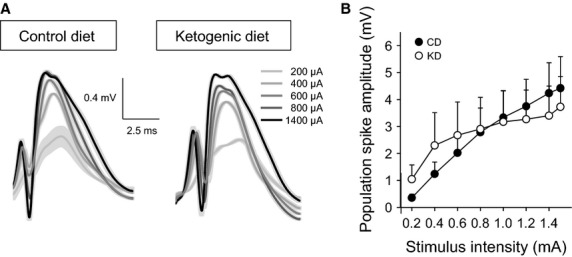
Input–output current-voltage curves from CD-fed and KD-fed animals. Population spike amplitude was determined in a 0.2–1.5 *μ*A range. (A) Representative examples of field postsynaptic potentials at selected current intensities from rats fed the CD or KD during quiet waking. Increasing current is indicated by darker grays. Traces are averages of five single traces; standard errors are indicated in lighter grays. (B) There was no significant effect of dietary treatment on the input–output curve. Diet *F*_(1,126)_ < 0.1, not significant; Diet-x-Current *F*_(7,126)_ = 1.8, not significant.

**Figure 2:**
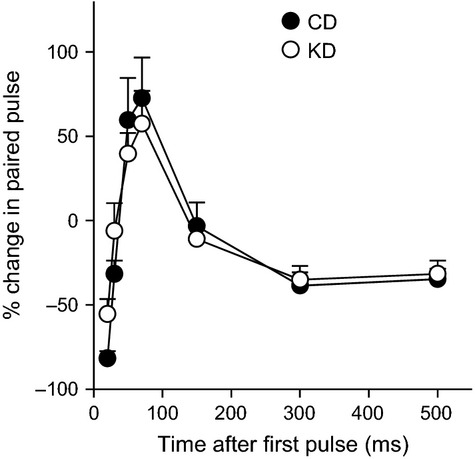
Paired-pulse analysis of short-term plasticity in CD-fed and KD-fed animals. The paired-pulse ratio was calculated over a 20–500 msec range. There was no significant effect of dietary treatment. Diet *F*_(1,108)_ < 0.1, not significant; Diet-x-Time *F*_(6,108)_ = 0.9, not significant.

After a 7-day postsurgical recovery, we performed chronic recordings successfully in 11 CD-fed and 9 KD-fed freely-moving juvenile rats (Fig.1A). Input–output current-voltage curves at the perforant path-dentate gyrus synapse were not significantly different in juvenile rats fed either diet (Fig.1B). Investigation of short-term plasticity using the paired-pulse protocol revealed the stereotypical depression/facilitation/depression pattern characteristic of this synapse when the interstimulus interval was increased over a range from 20 to 500 msec in both diet groups; there was no significant effect of dietary treatment (Fig.2).

**Figure 3:**
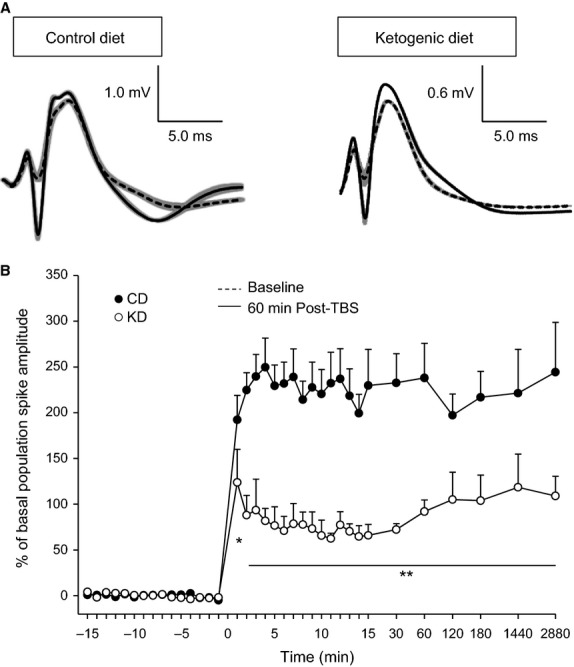
Long-term plasticity in CD-fed and KD-fed animals. (A) Representative examples of field postsynaptic potentials and superimposed population spikes recorded during quiet waking. Traces from baseline (dashed) and 60 min after (solid) *θ*-burst stimulation to induce LTP in rats fed the CD or KD. Baseline stimulation was calibrated to produce 50% of maximal population spike amplitude, and, using this same stimulation intensity, robust potentiation was produced by *θ*-burst stimulation. Traces are averages of five single traces; standard errors are indicated in gray. (B) *θ*-burst stimulation induced long-term plasticity over 48 h in CD-fed and KD-fed animals. Measurements were taken once every minute 15 min before and after induction (minute 0), and at decreasing frequency as time progressed and up to and including 48 h. There was a significant effect of dietary treatment in the amplitude of potentiation which remained until the last recording timepoint. Diet *F*_(1,803)_ = 9.1, *P* = 0.006; Diet-x-Time *F*_(35,803)_ = 6.6, *P* < 0.001. *P < 0.05, **P < 0.002 CD vs. KD.

After paired-pulse recordings across the interval range, and a subsequent 15 min baseline, long-term plasticity was investigated with a theta burst pattern of tetanic stimulation. Significant potentiation of synaptic transmission was immediate and robust in both dietary treatment groups (Fig.3), demonstrating that the KD did not prevent LTP induction. This significant potentiation remained stable throughout the recording period, and was reconfirmed as present and significant when rats were returned to the recording chamber and synaptic transmission was quantified at 24 and again at 48 h post-LTP induction. However, while synaptic transmission increased significantly compared to baseline, LTP magnitude was significantly lower in the KD-fed group; this reduction was apparent at all timepoints tested (Fig.3B).

## Discussion

Here, we found that the synaptic effects of a ketogenic diet were specific to the magnitude of long-term synaptic plasticity induced by theta-burst stimulation in freely-behaving juvenile rats. Similarly, in clinical use, KD feeding reduces seizure severity and frequency while having seemingly few effects on normative brain function (an effect consistent with the largely normal cognitive performance in nonepileptic individuals in most studies (Halyburton et al. 2007; Brinkworth et al. 2009; Krikorian et al. 2012). While these results contrast with a study in anesthetized juvenile rats that showed KD feeding did not alter hippocampal LTP (Thio et al. 2010), they are consistent with those from a previous study in awake, freely behaving adult rats (Koranda et al. 2011), highlighting the potential impact of anesthesia on synaptic plasticity. Together, our results in juvenile and adult rats suggest a common impact of KD regardless of age. Indeed, although most commonly used in treating pediatric epilepsy, KDs are also effective in adult epileptic patients (Baborka 1930; Sirven et al. 1999; Bodenant et al. 2008; Mosek et al. 2009; Klein et al. 2010). However, the effect of a KD may not be unidirectional and be homeostatic or restorative under some conditions. For example, KD feeding rescues dysfunctional hippocampal LTP in a murine model of multiple sclerosis (Kim et al. 2012), and also rescues hippocampal neurotransmission per se if impaired by hypoglycemia or respiration inhibition (Bough et al. 2006; Kim et al. 2010). This is perhaps not surprising given evidence for KD promotion of neuroprotection (Ruskin and Masino 2012), neural homeostasis (Boison et al. 2013), and normalized gene expression (Kobow et al. 2013; Woolf and Scheck 2015).

To mimic a KD in vitro, some groups have applied ketones. Exogenous ketones reduce synaptic glutamate release (Juge et al. 2010), and enhance K_ATP_ channel current (Tanner et al. 2011), mechanisms by which ketones could reverse hyperexcitable states and theoretically limit synaptic plasticity – suggesting, as above, that ketone-based metabolism may serve to normalize synaptic function. Also, exogenous ketones rescue hippocampal LTP during oxidative impairment (Maalouf and Rho 2008) and sustain neurotransmission during hypoglycemia (Izumi et al. 1998; Page et al. 2009). Yet KD feeding also chronically lowers circulating glucose, and recapitulating this effect by moderately lowering glucose in vitro (in the absence of exogenous ketones) reduces neuronal excitability via an adenosine-based mechanism (Kawamura et al. 2014). While ketone application paradigms can provide important information, acute ketone and/or glucose treatments are unlikely and perhaps unable to reproduce all of the acute and chronic effects of a diet – such as upregulated brain mitochondrial function associated with long-term KD treatment (e.g. Bough et al. 2006).

In the present study, KD-fed juvenile rats showed significant hippocampal LTP, and the population spike remained potentiated at least up to 48 h at a similar magnitude as at LTP induction. At all timepoints, however, the potentiation was lower in magnitude in KD-fed as compared to CD-fed rats, and consistent with results found in adult rats (Koranda et al. 2011). Taken together, these findings suggest KD feeding may reduce or limit LTP magnitude at the point of induction with no concomitant effect on mechanisms involved in LTP maintenance. If KD effects are limited to altering neuronal activity during the induction phase of LTP, that is, an epoch of strong excitation, this pattern is consistent with KD modulation of neuronal excitability preferentially during hyperexcitable states, for example, seizures. Consistent with this, in previous in vitro work, we found KD feeding limits epileptiform activity in CA3 of hippocampus by promoting ATP-sensitive K^+^ channel (K_ATP_ channel) activity (Kawamura et al. 2014). Others found that KD feeding elevates circulating polyunsaturated fatty acids that can activate K^+^ channels (Lauritzen et al. 2000; Börjesson et al. 2008; Xu et al. 2008), and that modifications of the protein BAD that mimic metabolic effects of the KD also decreased seizure susceptibility and required K_ATP_ channels (Giménez-Cassina et al. 2012). Consistent with this, pharmacological block of K_ATP_ channels augments LTP in CA1 of hippocampus (Schröder et al. 2004). Thus, while many acute and chronic mechanisms mobilized by a KD may contribute to its effects, it is plausible that one mechanism whereby KD feeding limits LTP in the dentate gyrus is by activating K_ATP_ channels.

The ideal LTP magnitude under different conditions remains unknown. However, mechanisms of LTP and epileptogenesis may overlap. Limiting LTP magnitude may be beneficial rather than detrimental in an epileptic brain – particularly if, as demonstrated here and by others – baseline synaptic transmission is unaffected. Supporting this point, the influence of KDs or other ketogenic treatments on learning, memory, and cognition seems to be beneficial across species – particularly in cases of neurological deficits and disabilities. For instance, in mouse models of Alzheimer's disease, diet supplementation with a medium-chain triglyceride (MCT, a fat easily metabolized to ketones) or a ketone ester (a ketone precursor) reduced learning and memory deficits (Aso et al. 2013; Kashiwaya et al. 2013), although no benefit was reported with a standard KD (Brownlow et al. 2013). A standard KD improved learning deficits in a mouse model of multiple sclerosis (Kim et al. 2012). In rats, ketones elevated by KD feeding correlate with protection against obesity-related cognitive impairments (Davidson et al. 2013). In aged dogs, feeding with MCTs aids cognition and learning (Pan et al. 2010). Notably, these strategies are also useful in aging patients: in studies including double-blind placebo-controlled designs, ketone-boosting strategies in patients with Alzheimer's disease or mild cognitive impairment significantly improved cognition and memory (Reger et al. 2004; Henderson et al. 2009; Krikorian et al. 2012; Newport et al. 2015). In all these studies, improvement was associated with elevated blood ketones. In epileptic patients, cognitive outcomes after KD treatment are also favorable (e.g. Lambrechts et al. 2013; Singh et al. 2014), possibly relating partly to seizure abatement. In epileptic patients with glucose transporter deficiency, a standard KD improved verbal fluency and delayed recall (Ramm-Pettersen et al. 2014), while some patients benefited cognitively from MCTs (Pascual et al. 2014). In neurologically normal subjects, two studies reported that long-term KD treatment improved cognition (Halyburton et al. 2007; Brinkworth et al. 2009), whereas another reported a mild and transient worsening on one of three cognition measures (Wing et al. 1995). Clearly, more studies of the effects of KDs and other ketogenic treatments on learning, memory, cognition, and the electrophysiological substrates of those processes – that is, synaptic plasticity – are warranted in diverse research models as well as normal subjects and those with various disorders.

## Conflict of Interest

The authors declare no conflicts of interest.
